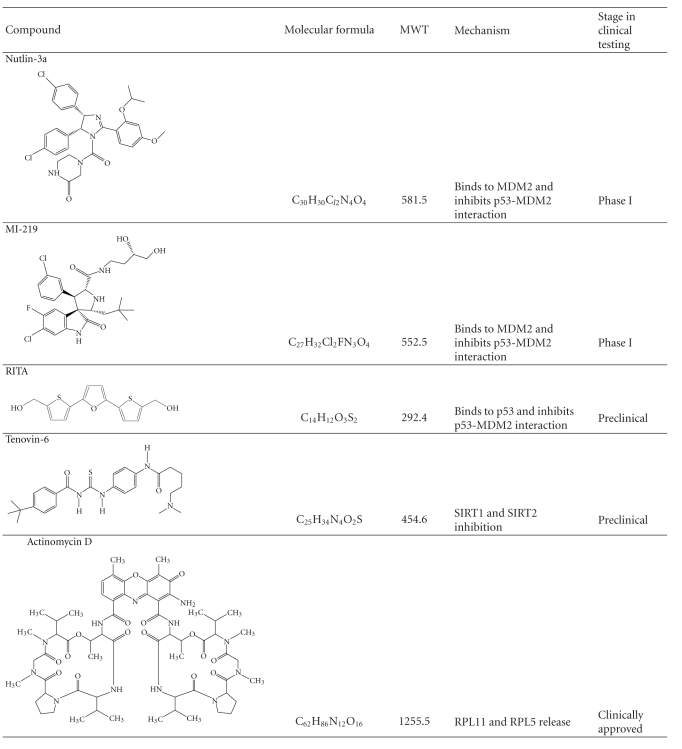# Targeting the p53 Pathway in Ewing Sarcoma

**DOI:** 10.1155/2011/746939

**Published:** 2010-12-09

**Authors:** Paul M. Neilsen, Kathleen I. Pishas, David F. Callen, David M. Thomas

**Affiliations:** ^1^Sarcoma Research Group, Discipline of Medicine, University of Adelaide and Hanson Institute, Frome Road, Adelaide, SA 5000, Australia; ^2^Ian Potter Foundation Centre for Cancer Genomics and Predictive Medicine, Peter MacCallum Cancer Centre, East Melbourne, VIC 3002, Australia

## Abstract

The p53 tumour suppressor plays a pivotal role in the prevention of oncogenic transformation. Cancers frequently evade the potent antitumour surveillance mechanisms of p53 through mutation of the *TP53* gene, with approximately 50% of all human malignancies expressing dysfunctional, mutated p53 proteins. Interestingly, genetic lesions in the *TP53* gene are only observed in 10% of Ewing Sarcomas, with the majority of these sarcomas expressing a functional wild-type p53. In addition, the p53 downstream signaling pathways and DNA-damage cell cycle checkpoints remain functionally intact in these sarcomas. This paper summarizes recent insights into the functional capabilities and regulation of p53 in Ewing Sarcoma, with a particular focus on the cross-talk between p53 and the EWS-FLI1 gene rearrangement frequently associated with this disease. The development of several activators of p53 is discussed, with recent evidence demonstrating the potential of small molecule p53 activators as a promising systemic therapeutic approach for the treatment of Ewing Sarcomas with wild-type p53.

## 1. Introduction

The p53 protein, known as the “guardian of the genome” [1] and voted Science magazine's “Molecule of the Year” in 1993, plays a pivotal role in the cellular defense against transformation of cells in the presence of oncogenic or genotoxic stress [2]. This is achieved through the ability of the p53 transcription factor to drive the expression of downstream target genes to evoke cellular responses such as cell cycle arrest, apoptosis, DNA damage repair, and senescence [3]. The development of a malignant neoplasm generally requires attenuation of these p53 responses, and this can occur via mutation of the p53 protein. *TP53* is the most frequently altered gene in cancer, with p53 mutations observed in approximately half of all tumours [4]. In contrast, *TP53* mutations are infrequent in the Ewing Sarcoma Family of Tumours (ESFTs) with the majority of these sarcomas expressing a functional wild-type p53 [5–14]. Such features are rarely seen in cancers and are suggestive that ESFTs will be sensitive to p53-based targeted therapeutic strategies.

## 2. Genomic Integrity of *TP53* Is Preserved in Ewing Sarcomas

Ewing Sarcomas arise in the bones of children and young adolescents and are the most lethal of all bone tumours [15, 16]. These sarcomas are infrequent neoplasms with international incidence rates in the pediatric population averaging less than two cases per million children. Such low incidence rates have limited the detection of *TP53* mutations to relatively small cohorts from independent clinical centres, with these studies reporting *TP53* mutation frequencies ranging from 4% to 20%. A meta-analysis of all primary or metastatic ESFTs involving *TP53* point mutations confirmed through direct sequencing reveals that *TP53* mutations are observed in approximately 10% of cases (Table 1). 

A similar analysis of the available literature suggests that the majority of other sarcoma types are also associated with low frequencies of *TP53* mutations, ranging from approximately 6% in well-differentiated/dedifferentiated liposarcomas to 23% in osteosarcomas (Table 2). Although malignant fibrous histiocytomas are listed, they are a discredited entity and will soon be removed from the World Health Organisation (WHO) sarcoma atlas [17]. Collectively, Ewing Sarcoma was associated with the lowest frequencies of *TP53* mutation across all sarcoma types.

The International Agency for Research on Cancer (IARC) has recently released recommendations for the detection of *TP53 *mutations, and advise direct sequencing of exons 4 to 10 of the *TP53 *gene [46]. Studies listed in Tables 1 and 2 rarely fulfilled these recommendations. Furthermore, the reported frequencies of *TP53* mutations may be marginally underestimated as the majority of these studies sequenced *TP53* in patients where an initial screen detected overexpressed p53 protein by immunohistochemistry, rather than performing an unbiased sequencing of all cases. Although mutant p53 is typically stabilized in cancer cells, and overexpression of p53 protein is predictive of *TP53 *mutation [47], this indirect approach cannot detect heterozygous truncating mutations of *TP53* [46]. Nevertheless, frequencies of *TP53* mutation reported by the studies in Tables 1 and 2 are consistent with publicly available sequencing data from the IARC *TP53* database in which *TP53* point mutations were observed in 373 out of 2145 (17.4%) tumours from bone or soft tissue origins [46]. 

The integrity of the p53 pathway in Ewing Sarcomas is further supported by studies suggesting that gross chromosomal alterations involving the *TP53* locus on chromosome 17p are relatively infrequent in ESFT samples [48]. This is in contrast to other bone malignancies, such as osteosarcomas, where chromosome alterations of the *TP53* gene are frequently observed [24, 44, 49, 50].

## 3. Genetic Alterations in Regulators of the p53 Pathway

Typically, cancers that retain wild-type p53 have been shown to indirectly suppress the p53 regulatory and signaling pathways. One of the most common oncogenic defects observed involves amplification or overexpression of MDM2 [51]. The stability and activity of p53 are constitutively regulated by MDM2 using an auto-regulatory feedback loop which in normal cells prevents inappropriate activation of p53 [52]. MDM2 is an E3 ubiquitin ligase that antagonizes the tumour suppressor function of p53 by silencing the ability of p53 to transactivate target genes or promoting its degradation or nuclear exportation [53, 54]. However, *MDM2* amplification is a rare event in Ewing Sarcomas and is only observed in approximately 2% of ESFT cases (Table 3). In contrast, virtually all well-differentiated and dedifferentiated liposarcomas contain complex marker chromosomes with multiple copies of the *MDM2* locus [55]. 

Cancers can also attenuate p53 function through deletion of *CDKN2A*, the gene encoding p14^ARF^ [48]. The p14^ARF^ tumour suppressor is a positive regulator of p53 in response to specific stimuli such as oncogenic stress. The stability of p53 is enhanced by p14^ARF^ through its ability to sequester MDM2, thus releasing p53 to activate downstream pathways of growth suppression or apoptosis [56, 57]. A summary of the available literature related to *CDKN2A* chromosomal alterations in ESFT cases revealed that either homozygous or hemizygous deletion of the *CDKN2A* locus are also relatively infrequent events, occurring in less than 20% of all cases (Table 3). *CDKN2A* alterations and *TP53* mutations are mutually exclusive events in the majority of ESFT cases, suggesting that either genetic insult is sufficient to inactivate the p53 pathway in these cancers [11, 48]. 

MDM4 is another key negative regulator of the p53 pathway [61–63]. This oncoprotein is closely related to MDM2 with significant homology between their DNA binding domains; however, MDM4 has a more specific role in the negative regulation of p53 transcriptional activity. Amplification of the *MDM4* gene has also been reported in several tumour types, with *MDM4* amplification observed in 65% of retinoblastomas [64]. *MDM2* and *MDM4* amplification are rarely observed within the same tumour, suggesting that either event is sufficient to inactivate the p53 pathway. *MDM4* amplification is a possible mechanism for functional inactivation of the p53 pathway in Ewing Sarcoma given the infrequent occurrences of *MDM2* amplification, *CDKN2A* deletion, or *TP53* mutation in these cancers. Unfortunately, previous cytogenetic studies are restricted to investigating allelic imbalance of the *TP53*, *MDM2,* and *CDKN2A* loci in ESFT patient material, hence the frequency of* MDM4* amplifications in Ewing Sarcoma is currently unknown. 

The small proportion of ESFT cases with either *MDM2* amplification or p14^ARF^ deletion cannot collectively account for the ability of Ewing Sarcomas to develop in a cellular context with wild-type p53. Further investigations of Ewing Sarcoma are warranted to conclusively determine if the mechanisms that attenuate the p53 response during sarcomagenesis occur at the genetic or posttranslational level. The presence of an *EWS-ETS* translocation event is a universal feature of Ewing Sarcoma and represents another possible genetic alteration responsible for the regulation of p53 in ESFT. Recent insights into the functional characterization of the resulting oncogenic gene product suggest a potential role of this ubiquitous translocation event in silencing p53 activity in ESFTs (see later discussion).

## 4. The p53 Signaling Pathways Are Functionally Intact in Ewing Sarcoma

Abrogation of the p53 pathway through *TP53* mutation is typically associated with enhanced tumour invasive and metastatic capabilities, and poorer patient survival rates [46, 65]. Ewing Sarcoma is an aggressive malignancy with the lowest patient survival rates of all primary musculoskeletal tumours, traits rarely possessed by cancers that retain wild-type p53. Despite observations that *TP53* alterations and *MDM2* amplifications are infrequent events in primary Ewing Sarcomas, it has been speculated that the downstream signaling pathways of p53 may be inactive in these sarcomas. In order to test the functional intactness of these p53 signaling pathways in Ewing Sarcoma, Heinrich Kovar and colleagues investigated the response of several ESFT cell lines with varying p53 status to ectopic p53 expression [66]. A prolonged apoptotic or growth arrest phenotype was observed upon ectopic expression of wild-type p53 in the cell lines. The sensitivity of Ewing Sarcoma cell lines to X-irradiation was also dependent on the expression of an endogenous wild-type p53. These findings confirm the intactness of the p53 signaling pathways in Ewing Sarcoma. 

The frequent normal functioning of the p53 signaling pathway in ESFTs is also demonstrated by the observation that almost all Ewing Sarcoma cell lines have acquired either *TP53 *mutations or *CDKN2A* deletions, suggesting selective pressure for these genetic alterations to permit *in vitro* growth [67]. Similar observations have been made in the clinic, with p53 mutation and *CDKN2A* deletion defining a lethal subset of ESFTs associated with poor response to chemotherapy [12]. In conclusion, the *in vitro* and *in vivo* evidence suggests the p53 signaling pathways are intact in a significant proportion of Ewing Sarcomas.

## 5. The Prognostic Significance of *TP53* Alterations

ESFT patients with point mutation of *TP53* are associated with a poor prognosis [12, 68, 69]. Logically, one would expect a genetic event that confers a growth advantage to be highly represented across a tumour type through selective pressure. This is not the case in Ewing Sarcoma, as *TP53* mutations rarely occur, yet define a high-risk population of patients. 

Huang and colleagues have provided the most compelling evidence thus far for *TP53* mutation as an independent prognostic marker using a combined immunohistochemistry, Genechip and sequencing approach to detect *TP53* mutations in 60 ESFTs [12]. *TP53* mutations were identified in 8 of these 60 cases (13.3%), and all eight patients expressing mutant p53 ESFTs died within 21 months of diagnosis with a mean survival of 11 months, as compared to a mean survival of 99 months for patients with wild-type p53 ESFTs. Multivariate analysis identified *TP53* mutation as the strongest independent prognostic factor [12]. This is the largest prognostic study to date that involves DNA sequencing of *TP53* in ESFT patient material. 

The prognostic involvement of p53 mutation in Ewing Sarcoma was recently challenged by findings from a retrospective study involving 308 ESFT cases collected from 1971 to 2007 [48]. Although overexpression of p53 protein was detected in 25% of these cases, this study restricted the classification of “p53 mutation” to these cases which did not express p21^Waf1/Cip1^, the strongest canonical p53 target. Subsequently, 15% of ESFT cases where deemed to express mutant p53 upon application of these criteria. This study showed that “mutant p53” expression was more frequent in disseminated disease than in primary localized tumors, indicating a role in the progression and metastasis of Ewing Sarcoma. However, there was no association between “mutant p53” expression and patient survival. The conclusions from this study are limited by the absence of actual *TP53* sequencing of ESFT samples to confirm the presence of *TP53* mutations. These observations need to be attested by further investigation into the prognostic value of *TP53* mutations using unbiased direct sequencing approaches.

Gross chromosome rearrangements involving *TP53* have been recently reported to influence the prognosis of Ewing Sarcoma [48, 70]. Alteration of 17p (the chromosomal arm containing the *TP53* locus) was observed in 16.7% of ESFT samples and was associated with significantly poorer survival rates [70]. Lopez-Guerrero and colleagues recently showed that alteration the *TP53* locus alone was a prognostic marker for poor patient outcome [48]. Gross alteration of *TP53* gene was detected in 32 of 191 (17%) ESFT cases. Interestingly, the strongest prognostic information from these studies was observed upon loss of heterozygosity (LOH) of 16q, which occurred in 20.8% of cases and was the most significant indicator of poor outcome [70]. A recent study that used a combined comparative genomic hybridization (CGH) and expression microarray analysis identified the *ANKRD11* locus at 16q24.3 as one of the most frequently deleted and down-regulated genes in Ewing Sarcoma [71]. It is noteworthy that ANKRD11 was recently reported as a p53 coactivator [72], suggesting that the loss of p53 activators may contribute towards the ability of Ewing Sarcomas to develop and progress in the presence of a wild-type p53. 

Studies to date are consistent with the presence of *TP53* point mutations in defining a high-risk population of ESFT patients. However, more detailed studies are warranted to conclusively evaluate the prognostic potential of *TP53* point mutation. Collectively, these findings indicate that the prognostic potential of mutant p53 will be fully realized through the application of definitive approaches to detect *TP53* mutations.

## 6. Oncogenic *EWS-ETS* Translocations

ESFTs are cytogenetically diagnosed through specific genetic rearrangement involving the *EWS* gene (official symbol *EWSR1*) and a member of the *ETS *transcription factor gene family. This chimeric fusion protein is present in over 90% of ESFTs [73, 74] and is widely considered to be causative of this malignancy. The *EWS-FLI1* translocation, t(11;22)(q24;12), is a chromosomal aberration specific to ESFTs and accounts for 85% of translocation events in Ewing Sarcoma. This reciprocal translocation generates fusion of the 5′ segment of *EWS* on chromosome 22 with the 3′ segment of *FLI-1* on chromosome 11. Antisense DNA studies have confirmed that continuous EWS-FLI1 expression is required for the *in vitro* proliferation and *in vivo* tumorigenic capacity of Ewing Sarcoma cells [75–78]. The second most common EWS translocation described involves an in-frame fusion of the *EWS* and *ERG *genes as a result of the t(21;22)(q22;q12) translocation, accounting for 5% of translocations in Ewing Sarcoma [74].

## 7. Suppression of p53 Activity by EWS-FLI1

Attenuation of the p53 tumour surveillance mechanisms during the development and progression of Ewing Sarcomas may be explained through the ability of the EWS-FLI1 oncoprotein to silence p53 activity. Two independent studies have shown that silencing of EWS-FLI1 expression in Ewing Sarcoma cell lines increases p53 activity [79, 80], suggesting that the EWS-FLI1 fusion protein plays a role in the constitutive silencing of p53 tumour suppressor activity. It appears that EWS-FLI1 can achieve this through either an indirect mechanism, involving the Notch signaling pathway [79], or through the formation of a protein complex involving EWS-FLI1 and p53 [80]. This study suggests that EWS-FLI1 attenuates p53 activity through physically sequestration facilitated by the EWS region of the fusion protein [80]. However, it is unclear whether interaction between p53 and EWS-FLI1 occurs directly or is mediated through other oncogenic binding partners. It is of great interest that the amino region of EWS enables the recruitment of p53 to EWS-FLI1, as this p53-binding region is present in almost all Ewing Sarcoma gene translocation events and numerous other translocation-based cancers (Table 4). Such observations suggest that these malignancies share a common mechanism involving EWS that may potentially involve the functional inactivation of p53.

The ability of EWS-FLI1 to suppress p53 activity in Ewing Sarcoma is reminiscent of the functional role of the oncogenic translocation product in synovial sarcomas. The presence of the SS18-SSX fusion protein as a result of the t(X:18)(p11.2;q11.2) translocation is a universal feature of synovial sarcoma [94]. These sarcomas abrogate p53 protein levels through its enhanced proteasomal degradation facilitated by SS18-SSX [95]. Reminiscent of Ewing Sarcoma, *TP53* mutations are rare events in synovial sarcomas (Table 1), suggesting that these cancers rely on the ability of the SS18-SSX fusion protein to abrogate the p53 response, facilitating oncogenic transformation in the presence of a functional, wild-type p53.

## 8. EWS-FLI1 Expression Stimulates the p53 Pathway in Normal Cells, Fibroblasts, or Nonmesenchymal Cells

The development of an animal model to investigate the oncogenic properties of EWS-FLI1 has been limited by the toxic effects associated with the expression of this potent fusion protein in primary cells [96]. Due to the absence of an adequate transgenic animal model for Ewing Sarcoma, experimental approaches have been restricted to forced expression of EWS-FLI1 in various cell lines. Introduction of EWS-FLI1 into primary human fibroblasts resulted in a growth arrest through stimulation of the p53 pathway [96]. Subsequent specific inhibition of p53 activity in these fibroblasts rescued the growth arrest phenotype, allowing EWS-FLI1 to promote anchorage-independent growth of these fibroblasts. Similar effects have been observed in mouse embryonic fibroblasts (MEFs) in which expression of EWS-FLI1 induced the p53-dependent growth arrest or apoptosis [97]. This apoptotic or growth arrest response was considered to be p53-dependent, as MEFs null for p53, p19^ARF^, or p16 were unaffected in response to EWS-FLI1 expression. Normal cells retaliate this aberrant oncogene expression by mounting a p53-based defense mechanism resulting in cellular apoptosis or senescence [98, 99]. Consideration of the responses elicited by EWS-FLI1 in normal cells therefore suggests this fusion protein in functioning as a potent oncogene and is reminiscent of the responses associated with elevated levels of the *MYC* or *RAS* oncogenes. 

The cellular response to ectopically expressed EWS-FLI1 varies in primary cells of different origin. Ewing Sarcomas are derived from mesenchymal progenitor cells (MPCs) [100], and forced expression of EWS-FLI1 in MPCs was shown to be stably maintained without growth arrest or apoptosis whilst inducing an gene expression profile similar to that of a Ewing Sarcoma, all in the presence of a functional, wild-type p53 [101–103]. Such findings raise questions surrounding the ability of primary MPCs to tolerate forced EWS-FLI1 expression without engaging a p53-dependent response to the oncogenic stress. Nevertheless, the mechanisms used by MPCs to abide the expression of EWS-FLI1 in the presence of wild-type p53, whilst such expression in other primary cell lines triggers a p53 response, remain largely unknown. 

Attempts have been made to investigate the function of EWS-FLI1 in a transgenic mouse model. While the conditional expression of EWS-FLI1 in mouse MPCs did not induce the formation of Ewing Sarcomas, this expression of EWS-FLI1 in mice was able to influence sarcoma development in the absence of p53 [104]. Conditional *TP53* deletion in mouse MPCs led to the development osteosarcomas with a median tumour onset time of 50 weeks from birth. However, when EWS-FLI1 was conditionally expressed in these p53-null MPCs, an accelerated tumour growth from a median time of 50 to 21 weeks was observed, with the histological phenotype of these malignancies shifting towards a more poorly differentiated sarcoma [104]. These data provide *in vivo* evidence to further support the cross-talk between p53 and EWS-FLI1 which is essential in primary MPCs for the development of Ewing Sarcomas.

## 9. IGF1R and p53 Signaling Pathways

The activity of insulin-like growth factor 1 receptor (IGF1R) is essential for tumour development and progression through the signaling of antiapoptotic and prosurvival pathways [105–107]. IGF1R is also often overexpressed at the cell surface of malignant cells and thus has emerged as an attractive therapeutic target in cancer. A role of the IGF signaling pathway in the development of Ewing Sarcoma is evident through the finding that silencing of the EWS-FLI1 oncoprotein showed upregulation and activation of IGFBP genes [108]. Ewing Sarcoma cell lines are highly sensitive to IGF1R inhibitors, especially in combination with conventional chemotherapy [16, 109]. The IGF1R antagonist, AMG 479, has shown promising results in the treatment of ESFTs in a phase I clinical trial, indicating that Ewing Sarcomas may be particularly sensitive to intervention of the IGF1R signaling pathway [110, 111]. However, these studies raise vital questions as to why striking responses are seen in some, but not all, patients treated with these agents.

IGF1R and p53 drive distinctly opposing biological outcomes, with a significant level of molecular cross-talk occurring between these two signaling pathways. Initial studies suggested that p53 retorts the antiapoptotic signaling of IGF1R through repression of *IGF1R* expression [112]. Further antagonism of IGF1R activity by p53 was demonstrated though the identification of insulin-like growth factor binding protein 3 *(IGF-BP3)* as a novel p53-regulated target gene [113]. Induction of *IGF-BP3* gene expression by p53 enhanced secretion of an active form of IGF-BP3 capable of inhibiting IGF1R mitogenic signalling. Thus, the IGF1R signalling pathway is functionally antagonised by wild-type p53. Recent evidence has also shown that IGF1R is degraded by MDM2 [114, 115]. Sequestration of MDM2 in the nucleus by high levels of mutant p53 may be a possible explanation for the high levels of IGF1R observed in some cancers [115]. 

Interestingly, pharmacological inhibition of IGF1R signalling reduces MDM2 translational synthesis, which in turn stabilizes p53 [116]. IGF1R signalling therefore regulates the p53 pathway. Hence, the overexpression of IGF1R and frequent retention of a functional, wild-type p53 present an opportunity of combined use of specific IGF1R inhibitors with activators of the p53 pathway.

## 10. Pharmacological p53 Activation As a Systemic Therapy for Ewing Sarcoma

Ewing Sarcomas provide a unique tumor type in which the majority of cases retain the functionally intact p53 pathways that are kept in check by either EWS-FLI1 or through another unknown mechanism. At present, there is no evidence of permanent suppression of the p53 pathway by specific mutation of critical components. Therefore, the most likely scenario involves abrogation of p53 function via a reversible, posttranslational mechanism. This provides unique therapeutic opportunities through intervention with small molecules that directly stabilize and activate endogenous intracellular p53. This concept was first demonstrated using Nutlin-3a, a small molecule antagonist of MDM2, which has shown antitumour activity *in vitro *and *in vivo* through activation of the p53 pathway in tumour cells that retain wild-type p53 [117]. Nutlin-3a antagonizes the p53-MDM2 interaction by blocking the p53-binding pocket of MDM2 and as a consequence there is a rapid stabilization and accumulation of p53 protein levels [117]. Promising results from several preclinical studies have clearly demonstrated the therapeutic potential of Nutlin-3a in a variety of tumour types expressing wild-type p53, including liposarcoma [118], rhabdomyosarcoma [119], osteosarcoma [117], synovial sarcoma [120], neuroblastoma [121], retinoblastoma [64], and leukemia [122–124].

We have recently investigated the potential of a p53-targeted therapeutic approach for the treatment of Ewing Sarcoma using Nutlin-3a. Interestingly, exposure of Ewing Sarcoma cell lines to Nutlin-3a resulted in a robust apoptotic phenotype [125]. Nutlin-3a induced apoptosis required the presence of a wild-type p53 and did not influence the growth of ESFT cell lines expressing mutant p53. These findings provide confirmation of the functionality of downstream p53 pathways in ESFTs retaining wild-type p53 and suggest that p53 activators will provide a novel molecular-based therapeutic for the majority of ESFTs. 

Due to its aggressive nature and early systematic spread, treatment of ESFT is highly challenging. Current treatment protocols for ESFT patients involve multiagent chemotherapy [15]. Prior to the introduction of combinational chemotherapy, 5-year survival for patients diagnosed with ESFT was less than 10% [126]. Since the introduction of intensive VACD-IE (vincristine, actinomycin D, cyclophosphamide, doxorubicin, etoposide, and ifosfamide) chemotherapy regimens, the current 5-year survival rates for patients with localised disease are ranging from 60 to 70% [15, 127]. Nevertheless, Ewing Sarcoma still has the lowest survival rates of any of the musculoskeletal tumours, with minimal improvements to patient outcomes observed over the last decade. Hence, there is an urgent need to develop targeted therapeutic approaches to augment the action of these cytotoxic agents. The integral role of p53 in the DNA damage response pathways stimulated by these genotoxic agents suggests that p53 activators such as Nutlin-3a may provide a novel approach to augment intensive chemotherapies. Indeed, Nutlin-3a demonstrated synergistic activity with numerous chemotherapies from the VACD-IE protocol in Ewing Sarcoma [125] and in rhabdomyosarcoma [119]. Such encouraging observations will require further evaluation by the conduct of clinical trials using p53 activators as systemic therapies in combination with the current chemotherapy regimens for the treatment of wild-type p53 sarcomas. 

The identification of additional drugs that work on the same principle as Nutlin-3a is an area of active research within the p53 community. The majority of reported p53 activators (Table 5) have been shown to rapidly stabilize and activate p53 protein levels through inhibition of the MDM2-p53 interaction. One such example is the recently identified MI-219, a highly selective MDM2 antagonist [128]. MI-219 interacts with the p53 binding pocket of MDM2 with a higher affinity and selectivity than Nutlin-3a and hence attains a more potent stimulation of the p53 pathway. MI-219 was observed to achieve p53-dependent antitumor activity without causing visible signs of toxicity or gross abnormalities in mice [128]. In addition, MI-219 exhibits highly desirable pharmacokinetic properties and is currently in early-phase clinical trials. Small molecule p53 activators can also function through direct interaction with p53, as demonstrated with RITA (Reactivation of p53 and induction of tumour cell apoptosis) [129]. Preclinical studies show that RITA can induce a nongenotoxic activation of p53 through inhibition of the MDM2-p53 interaction via direct interaction with N-terminal domain of p53. 

MDM2 antagonists have been demonstrated to elicit their most potent effects in cell lines where *MDM2* is amplified or overexpressed [118]. Since this genetic event is not observed in all tumour types, small molecules that activate p53 through alternative pathways have been developed. Lain and colleagues identified the Tenovins, a class of p53 activators that enhance the acetylation of p53 [130]. The mechanism of action of Tenovin-1 and the water soluble analog Tenovin-6 involve the direct inhibition of SIRT1 and SIRT2, two members of the sirtuin family of class III histone deacetylases responsible for the deacetylation of p53 [131–133]. It is widely accepted that acetylation is an indispensible modification of p53 that occurs during specific activation of the p53 pathway [134, 135]. Interestingly, Tenovin-6 was shown to repress the growth of cancer cells using *in vitro* and *in vivo* models through hyperacetylation of p53 [130]. These studies imply that pharmacological inhibition of sitrins is an effective approach for p53 activation. 

Although specific p53 activation in tumours is an attractive therapeutic approach, these recently developed small molecules are under investigation in preclinical or early-phase clinical trials. In an attempt to accelerate the implementation of p53 activators in the clinic, a study led by Choong and colleagues screened a library of clinically approved drugs and successfully identified actinomycin D as a compound which mimics the action of Nutlin-3a when administered at specific dosages [136]. Surprisingly, low doses of actinomycin D induced specific activation of the p53 pathway with cellular responses remarkably similar to that of Nutlin-3a. These concentrations of actinomycin D were also demonstrated to augment the cytotoxic actions of chemotherapeutic drugs in cancer cells with wild-type p53 [136]. As actinomycin D is an FDA-approved drug, these findings catalyze the immediate application of a p53-based targeted therapeutic approach in the clinic.

High doses of actinomycin D are associated with intercalation into the DNA and subsequent double-strand DNA breaks [137]. It is presently used in the clinic as a chemotherapeutic agent and is a component of the highly successful combination treatment for Wilm's tumour. The inclusion of actinomycin D in current multiagent chemotherapy regimes for Ewing Sarcoma is variable amongst different clinical centers and is often dependent on the age of the patient. Since the pharmacokinetic data available for actinomycin D is limited [138, 139], comparison between the required *in vivo* dosages of this drug with the low concentrations used *in vitro* which elicit specific activation of p53 remains a formidable challenge. Interestingly, Ewing Sarcoma cells are highly sensitive to actinomycin D *in vitro*, with potent antitumour activity observed within the ranges described as “low dose” specifically in Ewing Sarcoma cell line that retains wild type p53 [125]. Further studies are warranted to evaluate the potential of incorporation of low dose actinomycin D with the current standard of care for the treatment of patients with wild-type p53 ESFTs.

## 11. Conclusion

Ewing Sarcomas share the common genetic features including the universal presence of the *EWS-ETS* translocation and frequent retention of the wild-type p53 and its associated functional downstream pathways. Targeted exploitation of the p53 pathway holds great promise to enhance the activity of current ESFT treatment regimes and improve the currently poor survival rates associated with Ewing Sarcoma. Recent identification of the first clinically approved drug, actinomycin D, as a p53 activator has facilitated the translation of these targeted therapies into current ESFT treatment regimens. Low dose actinomycin D hold an exciting potential as a directed molecular-based approach to specifically activate wild-type p53 in ESFTs, and the organization of clinical trials currently in progress to attest the potential of this approach. In addition, complementation of these studies with direct *TP53* sequencing of ESFT material would identify either patients with wild-type p53 tumours most likely to benefit from p53-based therapies or the less frequent “high risk” population of ESFTs containing point mutations in the *TP53* gene.

## Figures and Tables

**Table 1:** *TP53* Mutations in Ewings Sarcoma confirmed by DNA sequencing.

Sarcoma type	Study	Method	Exons sequenced	Mutation frequency	*TP53 *mutation
*Ewing Sarcoma*	Kovar et al., 1993 [5]	PCR-SSCP Sequencing	4–8	2/37	C277Y, **R273C**
	Komuro et al., 1993 [6]	PCR-SSCP Sequencing	5–9	2/14	152(FS), G154V
	Hamelin et al., 1994 [7]	PCR-DGGE Sequencing	5–8	2/12	**R175H**,** R248W**
	Patiño-García and Sierrasesúmaga, 1997 [8]	PCR-DDGE Sequencing	5–8	1/5	**R273H**
	Radig et al., 1998 [9]	IHC PCR-SSCP Sequencing	4–8	1/24	Not Specified
	Tsuchiya et al., 2000 [10]	PCR-SCCP Sequencing	5–9	1/24	G154V
	López-Guerrero et al., 2001 [11]	Sequencing	5–8	3/19	C135F, A138D, P151R
	Park et al., 2001 [13]	PCR-SSCP Sequencing	4–9	3/35	K132M, C135S, Q287V
	Huang et al., 2005 [12]	IHC p53 GeneChip Sequencing	—	8/60	W146(STOP), M160L N239A, G244I, **R248Q** **R273F**, A276S, R342P
	Schaefer et al., 2008 [14]	Sequencing	5–8	2/17	C141Y, **R248W**

Total *TP53 *mutations in Ewing Sarcoma					25/247 (10.1%)

DDGE: Denaturing Gradient Gel Electrophoresis; IHC: Immunohistochemistry; PCR-SSCP: Polymerase Chain Reaction Single-Strand Conformational Polymorphism; FS: Frameshift; Mutations in bold indicate p53 “hotspot mutations.”

**Table 2:** *TP53* Mutations in sarcomas other than ESFTs confirmed by DNA sequencing.

Sarcoma type	Study	Method	Exons sequenced	Mutation frequency	*TP53 *mutation
*Liposarcoma*					
Well-differentiated/de-differentiated liposarcoma (WD/DDLPS)	Pilotti et al., 1997 [18]	IHC PCR-SSCP Sequencing	5–9	4/13	H179Y, R213(STOP) **R282W**, Gg>Gc (SS)
	Dei Tos et al., 1997 [19]	IHC PCR-SSCP Sequencing	5–8	1/14	S127F
	Schneider-Stock et al., 1998 [20]	PCR-SSCP Sequencing	4–8	0/8	—
	Schneider-Stock et al., 1999 [21]	IHC PCR-SSCP Sequencing	5–8	0/13	—
	Barretina et al., 2010 [22]	Sequencing, mass spectrometry-based genotyping	—	0/50	—

				Total *TP53* mutations	5/98 (5.1%)

Myxoid/Round cell liposarcoma	Pilotti et al., 1997 [18]	IHC PCR-SSCP Sequencing	5–9	1/6	Del nts 1506-1507 (STOP)
	Schneider-Stock et al., 1998 [20]	PCR-SSCP Sequencing	4–8	1/12	P128S
	Schneider-Stock et al., 1999 [21]	IHC PCR-SSCP Sequencing	5–8	3/19	H214L, P250T, **G245S**
	Oda et al., 2005 [23]	IHC PCR-SSCP Sequencing	5–9	5/77	Q167(STOP), H214Y V225A, C238Y, C242Y
	Barretina et al., 2010 [22]	Sequencing, mass spectrometry-based genotyping	—	0/21	—

				Total *TP53* Mutations	10/135 (7.4%)

Pleomorphic liposarcoma	Schneider-Stock et al., 1998 [20]	PCR-SSCP Sequencing	4–8	3/6	**R248Q**, E271(STOP) **R273C**
	Schneider-Stock et al., 1999 [21]	IHC PCR-SSCP Sequencing	5–8	2/9	**R248Q**, **R273C**
	Barretina et al., 2010 [22]	Mass spectrometry-based genotyping	—	4/24	C135F, T155I C>TT (SS), C>CT (SS)

				Total *TP53* mutations	9/39 (23.1%)

Undefined liposarcomas	Toguchida et al., 1992 [24]	PCR-SSCP Sequencing	2–11	1/4	AGgt>AGtt (SS)
	Leach et al., 1993 [38]	IHC Sequencing	5–8	3/13	Q144(STOP), N239S GGT>GAT (SS)
	Latres et al., 1994 [25]	PCR-SSCP Sequencing	2–9	5/25	H168R, H193Y, M246V **R248W**, 344(STOP)
	Castresana et al., 1995 [26]	PCR-SSCP Sequencing	5–8	1/4	V216A
	Nawa et al., 1999 [27]	PCR-SSCP Sequencing	5–8	1/9	T253A
	Das et al., 2007 [28]	IHC Sequencing	2–11	1/3	377(FS)

				Total *TP53 *mutations	12/58 (20.7%)

Total *TP53* mutations in liposarcomas					36/330 (10.9%)

*Rhabdomyosarcoma*	Felix et al., 1992 [29]	PCR-SSCP Sequencing	4–8	1/6	R213P
	Toguchida et al., 1992 [24]	PCR-SSCP Sequencing	2–11	0/4	—
	Latres et al., 1994 [25]	PCR-SSCP Sequencing	2–9	0/2	—
	Castresana et al., 1995 [26]	PCR-SSCP Sequencing	5–8	1/1	V218L
	Kusafuka et al., 1997 [30]	PCR-SSCP Sequencing	5–8	1/10	**R273H**
	Nawa et al., 1999 [27]	PCR-SSCP Sequencing	5–8	0/2	—
	Taylor et al., 2000 [31]	PCR-SSCP Sequencing	5–9	1/20	Del nt 1004-1017
	Takahashi et al., 2004 [32]	PCR-SSCP Sequencing	5–9	9/45	E204G, R209T, P223R M243T, **G245C**, N247D, R249G, C291Q, P295H
	Das et al., 2007 [28]	IHC Sequencing	2–11	1/4	D393N

Total *TP53* mutations in rhabdomyosarcomas					14/94 (14.9%)

*Synovial Sarcoma*	Toguchida et al., 1992 [24]	PCR-SSCP Sequencing	2–11	0/5	—
	Latres et al., 1994 [25]	PCR-SSCP Sequencing	2–9	0/8	—
	Schneider-Stock et al., 1997 [33]	IHC PCR-SSCP Sequencing	5–8	0/2	—
	Dei Tos et al., 1999 [34]	PCR-SSCP Sequencing	—	4/20	Not Specified
	Nawa et al., 1999 [27]	PCR-SSCP Sequencing	5–8	1/7	L194F
	Schneider-Stock et al., 1999 [35]	IHC PCR-SSCP Sequencing	5–8	2/19	P128L, **R248W**
	Oda et al., 2000 [36]	IHC PCR-SSCP Sequencing	5–9	9/49	C141Y, A159T,V173M I195F, R196Q, G199R R213(STOP), N235D C238Y
	Das et al., 2007 [28]	IHC Sequencing	2–11	5/7	9(STOP), A63P, S96C, P250T, P250T
	Barretina et al., 2010 [22]	Sequencing, mass spectrometry-based genotyping	—	0/23	—

Total *TP53* mutations in Synovial Sarcomas					21/140 (15.0%)

*Malignant Fibrous Histiocytoma*	Toguchida et al., 1992 [24]	PCR-SSCP Sequencing	2–11	2/13	R196(STOP), **R273H**
	Andreassen et al., 1993 [37]	CDGE Sequencing	5,7,8	3/12	V143M, Y163C, G244D
	Leach et al., 1993 [38]	IHC Sequencing	5–8	1/11	R158H
	Latres et al., 1994[25]	PCR-SSCP Sequencing	2–9	0/9	—
	Castresana et al., 1995 [26]	PCR-SSCP Sequencing	5–8	3/12	Not Specified
	Schneider-Stock et al., 1997 [33]	IHC PCR-SSCP Sequencing	5–8	2/15	Y220C, C277(STOP)
	Nawa et al., 1999[27]	PCR-SSCP Sequencing	5–8	5/15	Y126F, **R175H** R213(STOP), S241T **R248Q**
	Das et al., 2007 [28]	IHC Sequencing	2–11	2/11	P77Q, 213(FS)

Total *TP53* mutations in malignant fibrous histiocytoma					18/98 (18.4%)

*Leiomyosarcoma*	Andreassen et al., 1993 [37]	CDGE Sequencing	5,7,8	2/6	K132M, **R248W**
	Latres et al., 1994[25]	PCR-SSCP Sequencing	2–9	5/13	Y163C, Y163C, H214R, G266E, ATgg>ATag (SS Intron 5)
	Patterson et al., 1994 [39]	PCR-SSCP Sequencing	4–9	6/29	P151H, P152L, R158H V216M, C238F, V272M
	Castresana et al., 1995 [26]	PCR-SSCP Sequencing	5–8	1/1	Not Specified
	Miller et al., 1996 [40]	PCR-SSCP Sequencing	2–11	1/8	Q165(STOP)
	Hall et al., 1997 [41]	IHC PCR-SSCP Sequencing	5–8	3/21	K163E, T211I A nt Del codon 246
	Schneider-Stock et al., 1997 [33]	IHC PCR-SSCP Sequencing	5–8	0/7	—
	Nawa et al., 1999[27]	PCR-SSCP Sequencing	5–8	0/3	—
	Zhai et al., 1999 [42]	IHC Sequencing	5–8	9/21	V173M, Y205C, S215R, **R248Q**, **R249W**, **R273H**, A276D, E285D, S303I
	Miyajima et al., 2001 [43]	IHC PCR-SSCP Sequencing	5–9	8/13	A161T, D184N, T220C T220C, C238S, C238C **R273H**, G279V
	Das et al., 2007 [28]	IHC Sequencing	2–11	0/5	—
	Barretina et al., 2010 [22]	Sequencing, mass spectrometry-based genotyping	—	0/27	—

Total *TP53* mutations in leiomyosarcomas					35/154 (22.7%)

*Osteosarcoma*	Toguchida et al., 1992 [24]	PCR-SSCP Sequencing	2–11	14/76	46 (STOP), 112 (STOP), **R175H** H193Q, E221(STOP) 227(STOP), S241Y G244V, P250L, D259V **R273H**, D281H, D281N aaTG > ggTCG (SS)
	Andreassen et al., 1993 [37]	CDGE Sequencing	5, 7, 8	2/11	D281E, E286K
	Castresana et al., 1995 [26]	PCR-SSCP Sequencing	5–8	2/7	169(STOP), D281Y
	Miller et al., 1996 [40]	PCR-SSCP Sequencing	2–11	13/42	H179Y, E224D, 239(FS), **G245D**, **R248W**, **R248**, 248(FS), **R273H** **R273H**, A276P **R282Q**, **R282Q** **R282H**
	Patiño-García and Sierrasesúmaga, 1997 [8]	PCR-DDGE Sequencing	5–8	6/37	**R175H**, R196(STOP) P250F, N268S, **R273H** **R2735**
	Gokgoz et al., 2001 [44]	PCR-SCCP Sequencing	4–10	60/272	agTCC > aaTCC (SS) L43(STOP), L43(STOP), P47L, 73(FS), 73(FS), 83(FS), 83(FS) In-Frame Ins (GGT) Codon 107/108 ACGgt/ACGtt(SS), agTAC/aaTAC (SS), V172D, **R175H** ATGgt/ATGat(SS), R181P, R196(STOP) V197G, Del codon 202–206, E204(STOP), Y205C R213(STOP), Y220C Y220C, E221(STOP) 31bp Del (FS) Intron 6 to Exon 7 229(FS), M237I, M237I, M237I, C238GC238G, C238G 241(FS), C242Y, C242Y, **G245S**, **G245S** **R248W**, **R248Q**, **R248Q**, **R248Q**, **R248Q**, P250L, T256S 15 bp In-frame Del (codon 265) **R273H**, **R273H**, **R273H**, R280H, D281H, D281H D281N, D281N E285K, Del codon 296–303 298(FS) GAGgt/GAGct (SS) R337C, R342(STOP) E343(STOP)
	Overholtzer et al., 2003 [45]	PCR-SCCP PCR-LDR	5–8	12/32	V173G, V173M, **R175H**, Del codon 175, Y220C, E224D, V272M, **R273H**, **R273C**, **D281H** FS (Exon 6), FS (Del 17nt) Exon 5

Total *TP53* mutations in osteosarcomas					109/477 (22.9%)

CDGE: Constant Denaturant Gel Electrophoresis; DDGE: Denaturing Gradient Gel Electrophoresis; IHC: Immunohistochemistry; PCR-SSCP: Polymerase Chain Reaction Single-Strand Conformational Polymorphism; PCR-LDR: Polymerase Chain Reaction Ligase-Detection Reaction; FS: Frame shift; Del: Deletion; Ins: Insertion; SS: Splice Site. Mutations in bold indicate p53 “hotspot mutations”. Recurrences with *TP53* mutations have been omitted in studies that reported both the primary tumour and recurrence with the same mutation.

**Table 3:** *MDM2* amplification or *CDKN2A* deletion in Ewing Sarcomas.

Study	*MDM2 *amplification	Study	*CDKN2A *deletion
Kovar et al., 1993 [5]	0/17	Kovar et al., 1997 [5]	7/27^#^
Ladanyi et al., 1995 [58]	3/30	Wei et al., 2000 [59]	7/39^#^
Tsuchiya et al., 2000 [10]	0/24	López-Guerrero et al., 2001 [11]	4/19^#^
Park et al., 2001 [13]	0/35	Brownhill et al., 2007 [60]	6/42*
López-Guerrero et al., 2001 [11]	0/19	López-Guerrero et al., 2010 [48]	34/169*

Total MDM Amplifications	3/125 (2.4%)	Total CDKN2A Deletions	58/296 (19.6%)

^#^Homozygous deletion of *CDKN2A*

*Includes both homozygous and hemizygous deletions of *CDKN2A.*

**Table 4:** Involvement of the *EWS* gene in translocation-based malignancies.

Tumour type	Fusion gene	Translocation	Reference
*Ewing Sarcoma*	*EWS-FLI1*	t(11;22)(q24;q12)	[73]
	*EWS-ERG*	t(21;22)(q22;q12)	[81]
	*EWS-ETV1*	t(7;22)(p22;q12)	[82]
	*EWS-ETV4*	t(17;22)(q12;q12)	[83]
	*EWS-FEV*	t(2;22)(q33;q12)	[84]

*Acute Leukemia*	*EWS-CIZ1*	t(12;22)(p13;q12)	[85]

*Angiomatoid Fibrous *	*EWS-ATF1*	t(12;22)(q13;q12)	[86]
*Histiocytoma*	*EWS-CREB1*	t(2;22)(q33;q12)	[87]

*Clear-cell Sarcoma*	*EWS-ATF1*	t(12;22)(q13;q12)	[88]
	*EWS-CREB1*	t(2;22)(q33;q12)	[89]

*Desmoplastic Small Round Cell Tumour*	*EWS-WT1*	t(11;22)(p13;q12)	[90]

*Extraskeletal Myxoid *	*EWS-CHN1*	t(9;22)(q22–31;q11-12)	[91]
*Chondrosarcoma*	*EWS-NR4A3*	t(9;22)(q22;q12)	[92]

*Myxoid Liposarcoma*	*EWS-DDIT3*	t(12;22)(q13;q12)	[93]

**Table 5:** Chemical structures and proposed mechanisms of small molecule p53 activators.